# *Wolbachia* impacts microbiome diversity and fitness‐associated traits for *Drosophila melanogaster* in a seasonally fluctuating environment

**DOI:** 10.1002/ece3.70004

**Published:** 2024-07-22

**Authors:** Lucas P. Henry, Michael Fernandez, Scott Wolf, Varada Abhyankar, Julien F. Ayroles

**Affiliations:** ^1^ Department of Ecology and Evolutionary Biology Princeton University Princeton New Jersey USA; ^2^ Lewis‐Sigler Institute for Integrative Genomics Princeton University Princeton New Jersey USA; ^3^ Department of Biology, Center for Genomics and Systems Biology New York University New York New York USA

**Keywords:** *Drosophila*, microbiome, seasonality, *Wolbachia*

## Abstract

The microbiome contributes to many different host traits, but its role in host adaptation remains enigmatic. The fitness benefits of the microbiome often depend on ecological conditions, but theory suggests that fluctuations in both the microbiome and environment modulate these fitness benefits. Moreover, vertically transmitted bacteria might constrain the ability of both the microbiome and host to respond to changing environments. *Drosophila melanogaster* provides an excellent system to investigate the impacts of interactions between the microbiome and the environment. To address this question, we created field mesocosms of *D. melanogaster* undergoing seasonal environmental change with and without the vertically transmitted bacteria, *Wolbachia pipientis*. Sampling temporal patterns in the microbiome revealed that *Wolbachia* constrained microbial diversity. Furthermore, *Wolbachia* and a dominant member of the microbiome, *Commensalibacter*, were associated with differences in two higher‐order fitness traits, starvation resistance and lifespan. Our work here suggests that the interplay between the abiotic context and microbe–microbe interactions may shape key host phenotypes that underlie adaptation to changing environments. We conclude by exploring the consequences of complex interactions between *Wolbachia* and the microbiome for our understanding of eco‐evolutionary processes that shape host‐microbiome interactions.

## Graphical abstract

Using field mesocosms, we performed longitudinal sampling to study microbiome dynamics in *Drosophila melanogaster* with and without *Wolbachia* in a seasonally changing environment. *Wolbachia* infection shaped patterns of microbiome diversity and interacted with a dominant microbe, *Commensalibacter*, to shape higher‐order fitness traits. Our results highlight the importance of understanding the influence of microbe–microbe interactions for host populations in a seasonally changing environment.

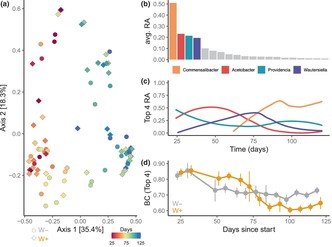

## 1 INTRODUCTION

The microbiome shapes many different traits in many different eukaryotic hosts, contributing to behavioral, metabolic, and immunological phenotypes (Henry et al., 2021; Johnson & Foster, 2018; McFall‐Ngai et al., 2013). While progress has been made in identifying the functional effects of the microbiome in laboratory settings, the eco‐evolutionary forces that generated the links between host and microbiome remain poorly understood (Henry et al., 2021; Koskella et al., 2017). One key reason is that the phenotypic effects and the potential fitness benefits of the microbiome on their host often depend on the local environment (David et al., 2018; Henry et al., 2021); changing environments can shift the relative costs and benefits of host–microbe interactions. Furthermore, the microbiome itself is dynamic. Feedback between the host and the environment can also change the composition and function of the microbiome (Carmody et al., 2021; Foster et al., 2017). The dynamic nature of the microbiome may itself be a key feature of host‐microbiome interactions, contributing to buffering the effects of environmental stress and potentially conferring key adaptive benefits for the host.

Transmission fidelity can also influence the evolutionary importance of the microbiome (Bruijning et al., 2021; Henry et al., 2021; van Vliet & Doebeli, 2019). Transmission fidelity refers to how faithfully the microbiome is shared across generations, between parents and offspring. Generally, for the microbiome to influence host fitness, microbes benefit their hosts, and hosts faithfully transmit the beneficial microbes to the next generation (Henry et al., 2021; van Vliet & Doebeli, 2019). Hosts can evolve strict control of microbial transmission through vertical transmission to maintain these beneficial interactions across host generations (Bruijning et al., 2021), such as the intricate molecular mechanisms that govern classic symbioses (e.g., the aphid‐*Buchnera* association (Koga et al., 2012)). However, strict control can limit the acquisition of other potentially more beneficial microbes, constraining hosts and microbes to the ecological conditions that generated the associations in the first place (Bennett & Moran, 2015; Henry et al., 2021; Kiers et al., 2010). Recent theoretical advances suggest that for organisms that occupy habitats with variable environments (e.g., seasonality or anthropogenic change), lower transmission fidelity and increased flexibility in the microbiome through environmental acquisition may actually benefit hosts (Bruijning et al., 2021). If the microbiome provides functions that benefit hosts, then flexibility in the microbiome may help hosts better match phenotypes to changing environments. Notably, the flexibility in the microbiome may also depend on microbe–microbe interactions. Vertically transmitted microbes are often present at embryogenesis, while other environmentally acquired microbes colonize throughout different points, over development and throughout the lifespan of hosts (Bright & Bulgheresi, 2010). Priority effects by the vertically transmitted microbes may thus facilitate or impede variation in the environmentally acquired microbiome (Debray et al., 2022; Sprockett et al., 2018), but the fitness effects of priority effects on hosts remain poorly characterized.

The microbiome of *Drosophila melanogaster* is an ideal system to test the fitness effects of interactions between vertically transmitted and environmentally acquired microbes. *D. melanogaster* harbors a common vertically transmitted bacterium, *Wolbachia pipientis*. *Wolbachia* is prevalent in *D. melanogaster*, infecting ~30% of the *Drosophila* Stock Center (Clark et al., 2005) as well as many natural populations (Hague et al., 2021; Riegler et al., 2005). The environmentally acquired microbiome in *Drosophila* microbiome is relatively simple (<20 species), environmentally acquired, and shapes many different traits (Douglas, 2018). Previous research suggests that *Wolbachia* has conflicting effects on the microbiome, with both antagonistic (Simhadri et al., 2017) and beneficial (Ye et al., 2017) effects on *Acetobacter* and *Lactobacillus*, the bacteria frequently observed in the fly microbiome. These bacteria are also implicated in shaping adaptation in *Drosophila*. In a field mesocosm experiment, inoculation with *Acetobacter* and *Lactobacillus* rapidly generated genomic divergence within five generations during fly adaptation to a seasonally changing environment (Rudman et al., 2019); however, fly populations harbored *Wolbachia*. In the laboratory, a meta‐analysis of experimental evolution in *Drosophila* found that *Wolbachia* and microbial diversity frequently responded to artificial selection (Henry & Ayroles, 2021), suggesting that the interactions between *Wolbachia* and the microbiome may contribute to host evolution. Indirect effects of *Wolbachia* may also be important, as *Wolbachia* has many impacts on *Drosophila* biology, shaping development and metabolism across the fly life cycle (Lindsey et al., 2021, 2023; Strunov et al., 2022). Furthermore, the effects of *Wolbachia* are often context‐dependent on both host genotype and ecological conditions (Harcombe & Hoffmann, 2004; Serga et al., 2021). Thus, *D. melanogaster* is an excellent model to study the fitness‐associated consequence of the interplay between host, microbiome, and the environment.

Here, using field mesocosms, we performed longitudinal sampling to study the microbiome dynamics in *D. melanogaster* with and without *Wolbachia* in a seasonally changing environment. In *D. melanogaster*, populations often undergo genomic and phenotypic change over their growing season during the transition from summer to fall (Grainger et al., 2021; Rudman et al., 2019, 2022). If bacteria with high transmission fidelity (i.e., *Wolbachia*) shape the microbiome and fitness effects on the host, then *Wolbachia*‐infected (W+) flies may differ in seasonal changes compared to *Wolbachia*‐free (W−) flies. We combined our longitudinal microbiome dynamics with phenotyping for fitness‐associated traits, starvation resistance at three timepoints towards the end of the season as well as lifespan at the end of the season to understand how *Wolbachia* and microbiome interactions influence host adaptation in a seasonally changing environment.

## 2 MATERIALS AND METHODS

### 2.1 Fly populations

Flies in this experiment were derived from a round‐robin crossing design of the Global Diversity lines (Grenier et al., 2015) and maintained at large population size (>10,000) flies for >100 generations before the field experiment. This base population was naturally infected with *Wolbachia* (W+). To generate the *Wolbachia*‐free (W−) population, flies were treated with 0.25 mg/mL tetracycline in the diet for two generations (Fry et al., 2004; Henry & Newton, 2018; Singh, 2019; Teixeira et al., 2008). For the initial tetracycline treatment, an egg lay to maintain the base W+ population was performed on the normal diet. Then, the next day, an egg lay using the same population was performed using the tetracycline diet to generate the W− population. We did not notice any substantial differences in egg density between the normal and tetracycline diets. After this initial egg lay, W− population was exposed for one more generation to the tetracycline diet. Following this, both W+ and W− populations were maintained in parallel on the standard diet. While we noticed some modest changes in the W− population (slightly lower density, slightly slower development) during the two rounds of tetracycline treatment, as others have shown that tetracycline can affect mitochondrial function and sex ratios in *Drosophila* (Ballard & Melvin, 2007; O'Shea & Singh, 2015). However, once the W− population was returned to the standard diet without antibiotics, there were no obvious differences in egg‐laying behavior, development, or population size between W− and W+ populations. W− flies were maintained for ~10 generations on the standard, no antibiotic diet before the beginning of this experiment at a similar population density as the W+ flies. During this pre‐experimental maintenance phase, all flies were maintained at 25°C with 12‐h light:dark cycles. All flies, in the lab and field, were reared on a diet composed of 10% glucose, 10% yeast, 1.2% agar with 0.04% phosphoric acid, and 0.4% propionic acid as preservatives.

To confirm *Wolbachia* status, we amplified two genes: cytochrome oxidase I (COI) in *D. melanogaster* (Nunes et al., 2010) and 16S rRNA gene from *Wolbachia* (O'Neill et al., 1992). The COI gene served as a positive control as all fly samples should always generate an amplicon. Primer sequences can be found in Appendix S1: Table M1.

### 2.2 Field design and experimental sampling

**FIGURE 1:**
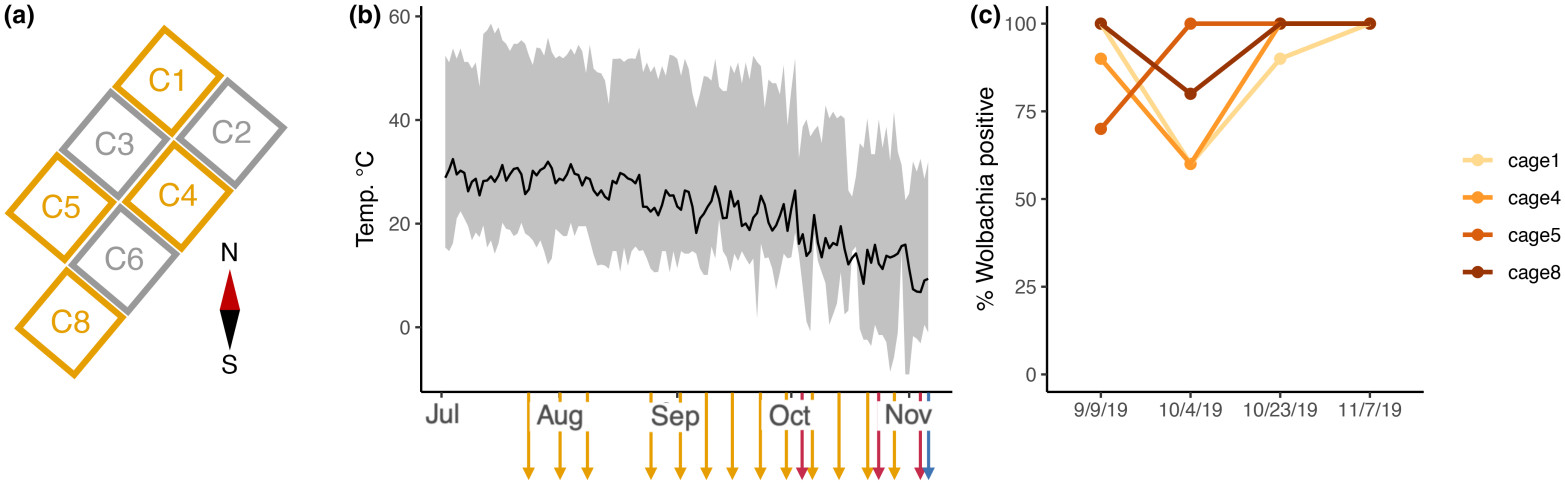
Experimental design. (a) Layout of the cages, with compass showing North–South orientation. Cages are colored by *Wolbachia* status, with gray representing *Wolbachia*‐free (W−) and orange representing *Wolbachia* (W+) flies. All cages maintained the initial *Wolbachia* status, except for C7, which was removed from all analyses. (b) Temperature and sampling regime over the season. Daily mean temperature is shown in black line and range shown in gray. The colored arrows represent sampling points. Microbiome was sampled weekly (orange, *N* = 13 timepoints) beginning Day 24 (third week of July) until Day 120. Flies were periodically phenotyped for starvation resistance as a proxy for fitness (red, *N* = 3 timepoints). Finally, at the end of the experiment (blue), lifespan was measured in females to test if *Wolbachia* status and microbiome variation influenced seasonal evolution. (c) Frequency of *Wolbachia* infection, measured in 10 individual females from each cage at four different timepoints. Colors represent the different W+ cages; all W− cages were 0% positive for *Wolbachia*.

The field site was located at Princeton University, NJ (40.34° N, 74.64° W). Eight total cages were constructed at the field site. Cages were 1.2 m × 0.6 m × 0.6 m (height × width × depth) and constructed from polyethylene monofilament fabric with 150 × 150 μm mesh (Greenhouse Megastore IS‐NT‐99). Each cage held a temperature/humidity data logger (Elitech USA Temlog 20H) placed on shelving units within the cage that held fly food. Depending on the position in the field, some temperature loggers may have been in direct sunlight during the daytime, but *Wolbachia* treatment was alternated across cages to deal with variation in sun exposure (Figure 1). While there was variation in temperature profiles for the different cages (Appendix S1: Figure M1), no significant difference in temperature was detected between W+ and W− cages (model: temperature ~ *Wolbachia* + Date + (1|Cage); *β*_WOLBACHIA_ = 0.138 ± 0.819 SE, *F* = 0.0284, *p* = .8744).

Approximately 2500 flies (equal sex ratio) were placed into each cage at the start of the experiment on July 2–3, 2019. We introduced 1000 flies on July 2 and an additional 1500 on July 3. We maintained populations by providing ~300 mL fly food once per week, allowing for flies to feed, lay eggs, and providing a substrate for larvae to develop. As the population grew, we provided food twice a week, with only one loaf pan/week kept with the developing flies to maintain population size. The other loaf pan was discarded before larvae reached the third instar and replaced with fresh fly food. Generations were overlapping, but we estimate that ~10 fly generations occurred during the experiment from July 2 until November 7, 2019. We initiated eight cages (4 infected with Wolbachia, 4 without Wolbachia), and all except one cage maintained its *Wolbachia* status throughout the course of the experiment (Appendix S1: Figure M2): Cage 7 was initially *Wolbachia*‐free, but we detected *Wolbachia* on the Day 57 sampling and subsequently at the rest of the sampling points. Cage 7 was removed from all analyses.

We allowed fly populations to stabilize for the first 3 weeks (~1–2 fly generations). Following this, we sampled flies approximately weekly to check *Wolbachia* status and characterize change in the fly microbiome. We note that the flies collected here were not specifically age‐matched but represent the standing variation of the microbiome within each cage at each timepoint. One pool of 10 flies was collected from each cage and PCR confirmed for *Wolbachia* status as previously described and then saved for 16S rRNA amplicon sequencing. One sampling point did not yield 16S rRNA sequencing results from all cages (August 7, 2019). This resulted in 13 timepoints over the season (Figure 1), starting with the first collection on July 24, 2019, and the final microbiome profile on October 28, 2019. The experiment concluded on November 7, 2019, when flies were collected for phenotyping.

During our weekly sampling, we tested for the presence of *Wolbachia* only in pools. To assess the frequency of *Wolbachia* infection within a population during the season, we tested 10 individual, age‐matched females (5–7 days old) at four timepoints from all cages: Day 71, Day 96, Day 116, and the final day, Day 127. Day 127 flies were not specifically age‐matched but collected from within the larger cage. *Wolbachia* infection was assessed using PCR as described above, and the relative frequency was compared across cages and these four sampling points. To generate age‐matched flies, we performed a separate egg lay using ~80 mL of food poured into a 2.5″ aluminum muffin tin (Reynolds). The density of eggs was monitored until it matched the approximate density for the larger egg lays, and then the muffin tin was put inside a smaller cage (8″ × 8″ × 8″). The larvae developed within the cage at the same temperature as the whole population. Then, once the flies began to eclose for 2 days, we removed the old food and placed new food in the muffin tin to allow the flies to age for 5–7 days post‐eclosion.

To understand how *Wolbachia* and the microbiome affected fly fitness, we collected flies for analysis of starvation resistance on three dates: Day 96, Day 116, and Day 127. To age‐match flies, we performed a separate egg lay in a cage within the cage as described above. Flies were age‐matched to 5–7 days old. For Day 116, we only were able to collect sufficient flies from two W+ cages. We note Day 127 flies were not specifically age‐matched, but collected from within the larger cage, which reflects the standing variation at the end of the season.

To measure starvation resistance, we video recorded flies in 15 × 6.25 mm (diameter × height) acrylic arenas over 4–5 days until death. One fly was placed per arena. There were 24 arenas within a plate, and each plate contained both sexes from two cages. Flies were aspirated into the plates to avoid side effects from CO_2_ anesthetization on behavior (Bartholomew et al., 2015). Arenas contained 1% agar in the bottom of the arena to provide humidity, but no nutritional value. Cages were randomized across observation plates. After plating flies, we recorded their movements at 1 frame per second using three Basler acA3088‐57um cameras allowing for full frame videos at 3088 × 2064. This yields approximately 8.197 px/mm – sufficient resolution to robustly identify individuals and their movements. All recordings were taken with LoopBio's Motif and compressed with libx264. After all the flies died, we determined the time of death from the video recordings. We determined the time of death manually in 2.5‐h intervals to quantify starvation resistance.

For the Day 127 flies, we also measured lifespan from individual females to identify whether this fitness‐associated trait varied between *Wolbachia* status at the end of the season Individual females were placed into fly vials with 6 mL fly food for ~24 h to lay and then flipped into a fresh vial. Flies were in this vial for 11 days. After this, flies were flipped every 3–4 days until death to determine the lifespan of each individual. We considered the age of the fly to be the time since we collected it in the field.

### 2.3 Microbiome profiling

The microbiomes were profiled using 16S rRNA amplicon sequencing. DNA was first extracted from pools (10 flies/pool for each cage every week, *N* = 13 timepoints) using the Quick‐DNA Plus kit (Zymo D4068), which includes a proteinase K digestion to ensure unbiased sampling of diverse bacteria. Samples were initially homogenized in lysis buffer and then split for DNA extraction with or without proteinase K digestion following the manufacturer's protocol. Proteinase K digestion did not affect the characterization of the microbiome (Appendix S1: Figure M3), thus in our analysis, we computationally merged samples from the same sampling point with +/− proteinase K. 16S rRNA amplicons were generated using a two‐step dual‐indexed approach. We amplified the V1–V2 region of the 16S rRNA gene (Appendix S1: Table M1), pooled for cleanup with Ampure XP beads, and then digested with BstZ17l enzyme to deplete *Wolbachia* amplicons (Simhadri et al., 2017). Libraries were sequenced using 300 bp paired‐end reads using the Illumina MiSeq platform at the Princeton University Genomics Core.

Sequences were processed using QIIME2 v2020.6 (Bolyen et al., 2019). DADA2 was used to cluster the amplicon sequence variants (ASVs) (Callahan et al., 2016). Taxonomy was assigned using the Greengenes reference database (DeSantis et al., 2006), trimmed to the 16S rRNA V1–V2 region. Phyloseq was used to visualize data (McMurdie & Holmes, 2013). Potential contaminants were flagged using the decontam package (Davis et al., 2018) and removed prior to analyses. Samples were rarefied to 1000 reads per pool for analyses (Appendix S1: Figure M4 for rarefaction).

### 2.4 Statistical analyses

To determine if *Wolbachia* altered the capacity for the microbiome to change during the seasonally fluctuating environment, we first calculated alpha diversity. We calculated Shannon diversity and Faith's phylogenetic diversity on ASVs. We first assessed if alpha diversity measures across all samples were impacted by *Wolbachia* status using a *t*‐test for Shannon diversity. We used the Kruskal–Wallis test for Faith's phylogenetic diversity because values were not normally distributed. We then calculated the change for both diversity measures from the prior sampling point and summed the absolute value of change. As we hypothesized that W+ flies would have lower change in diversity than W− flies, we tested for significance using a one‐sided *T*‐test. To further confirm that *Wolbachia* infection impacted change in alpha diversity, we used generalized additive models (GAM) implemented in mgcv (Wood, 2001). The model included *Wolbachia* infection status as a categorical term and time as smoothed term using cubic regression splines, with cage as the random effect. Faith's phylogenetic diversity was log_10_ transformed for normality of residuals.

For beta diversity, we first examined differences across all cages at all timepoints. We calculated Bray–Curtis dissimilarity (BC) for all samples and then used PERMANOVA implemented in vegan (Oksanen et al., 2015) to test for the effects of *Wolbachia* and time (over the course of the growing season) on community structure. To better understand how beta diversity changed over time, we then examined beta diversity change within each cage. We determined the change in BC with both the complete community and only the top four most abundant bacterial genera: *Acetobacter*, *Commensalibacter*, *Providencia*, and *Watuersellia*; four genera together comprised 83.1% bacterial reads across all samples. For the analyses of the top genera, all ASVs assigned to each genus of interest were included. The comparison between the complete community and the top four abundant bacteria allowed us to understand whether dominant microbes interact more with *Wolbachia* than low‐abundance bacteria. To further explore the effects of dominant microbes, we also tested if adding additional bacteria from the top 10 most abundant taxa influenced our results. We sequentially added the next most abundant bacteria and performed the same beta diversity analysis within cages. For example, after the top four, *Morganella* was the fifth most abundant bacteria. We filtered the community to include these top five most abundant bacteria and then performed similar analysis on BC change within cages. We repeated this analysis to include the top 10 bacteria. We used a mixed linear model to test for the effects of *Wolbachia*, time, and their interaction on log_10_‐transformed BC, with cage as a random effect, implemented in lme4 in R (Bates et al., 2007). To compare the effects of including more taxa, we examined the variance explained by the main effects estimated using MuMIn (Barton, 2009).

To assess the phenotypic effects of *Wolbachia* and microbiome interactions, we used *Commensalibacter* as a covariate in the starvation resistance analyses. *Commensalibacter* was the most abundant occurring bacteria for the three phenotyping timepoints, and the only bacteria found in all cages (Appendix S1: Figure M5). Given that the starvation resistance assay itself would alter the microbiome, we could not directly assess the microbiome of the flies we phenotyped. However, we used the *Commensalibacter* value from the preceding microbiome sample (i.e., Day 92 microbiome for the Day 96 phenotyping point). In essence, we ask if the microbiome preceding the phenotyping point is potentially predictive of phenotypic variation. For starvation resistance, we fit a mixed effect Cox proportional hazard model implemented in the coxme package in R (Therneau, 2015) for each timepoint separately. We modeled the response of starvation resistance (i.e., time to death) considering the fixed effects of *Wolbachia*, sex, *Wolbachia*  × sex interactions, *Commensalibacter* relative abundance, with cage, camera, and arena nested within imaging plate as random effects. Assumptions of proportional hazards were checked using Schoenfeld residuals. To confirm that using *Commensalibacter* was representative of microbial dynamics, we calculated Bray–Curtis dissimilarity for each of the three timepoints separately. PCoA Axis 1 was correlated with the relative abundance of *Commensalibacter* at each of the timepoints (Appendix S1: Figures M6–M8), and thus suggests that *Commensalibacter* captures the dominant differences in the microbiome.

To determine whether *Wolbachia* and the microbiome influenced fly fitness at the end of the sample season, we fit a mixed effect Cox model for longevity. As above, we used the preceding microbiome sample as we could not directly assess the microbiome of the phenotyped flies. We modeled the response of lifespan considering the effects of *Wolbachia* and *Commensalibacter* relative abundance, with cage and fly tube as a random effect, implemented in the coxme package in R (Therneau, 2015). Assumptions of proportional hazards were checked using Schoenfeld residuals. As for starvation resistance, *Commensalibacter* relative abundance was correlated with PCoA Axis 1 (Appendix S1: Figure M8).

## 3 RESULTS

### 3.1 Season shapes the composition of the microbiome

Flies were sampled weekly over the season beginning 3 weeks after the experiment started in July 2019. The frequency of *Wolbachia*‐infected flies decreased following the summer, but increased to 100% at the end of the growing season (Figure 1c). We note that while *Wolbachia* is part of the microbiome, for simplicity, we will refer to the microbiome as the bacterial community that primarily infects the gut and can also survive outside of the host (Douglas, 2018).

**FIGURE 2:**
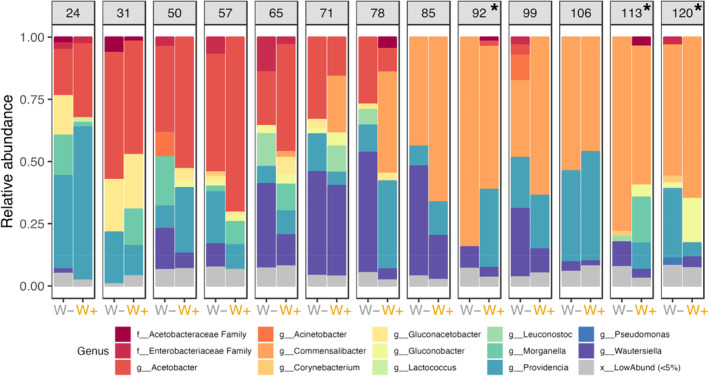
Microbiome composition over growing season. Groups are faceted by sampling date with averaged *Wolbachia*‐free (W−) and *Wolbachia* (W+) populations. Asterisks denote the dates that were paired with the fitness‐associated phenotyping later in the season. Colors represent the different genera.

The microbiome was predominantly composed of four bacteria: *Acetobacter*, *Commensalibacter*, *Providencia*, and *Wautersiella* (Figure 2). At the start of the season, *Acetobacter* and *Providencia* were the dominant bacteria. *Acetobacter* peaked early in the season (Day 57), and then was replaced by another bacterium in the Acetobacteraceae family, *Commensalibacter*, which dominated for the rest of the season. *Providencia* fluctuated, with peaks at the beginning and end. *Wautersiella* peaked in the middle of the season (Day 85), but was largely absent at the beginning and end. W− and W+ flies generally harbored the same bacteria, but the relative abundance differed over the season in complex ways; differences between *Wolbachia* status varied from one timepoint to the next.

### 3.2 *Wolbachia* constrains microbiome diversity in seasonally changing environment

If vertically transmitted microbes constrain the ability of the microbiome to respond to environmental fluctuations, then *Wolbachia* infection could reduce microbial diversity in two ways. First, *Wolbachia* infection could reduce the complexity of the community within a population (i.e., alpha diversity). Second, *Wolbachia* infection may change how community turnover proceeds over the season (i.e., beta diversity). Through longitudinal sampling across replicated W+ and W− populations, we assessed how *Wolbachia* infection shaped microbiome dynamics.

**FIGURE 3:**
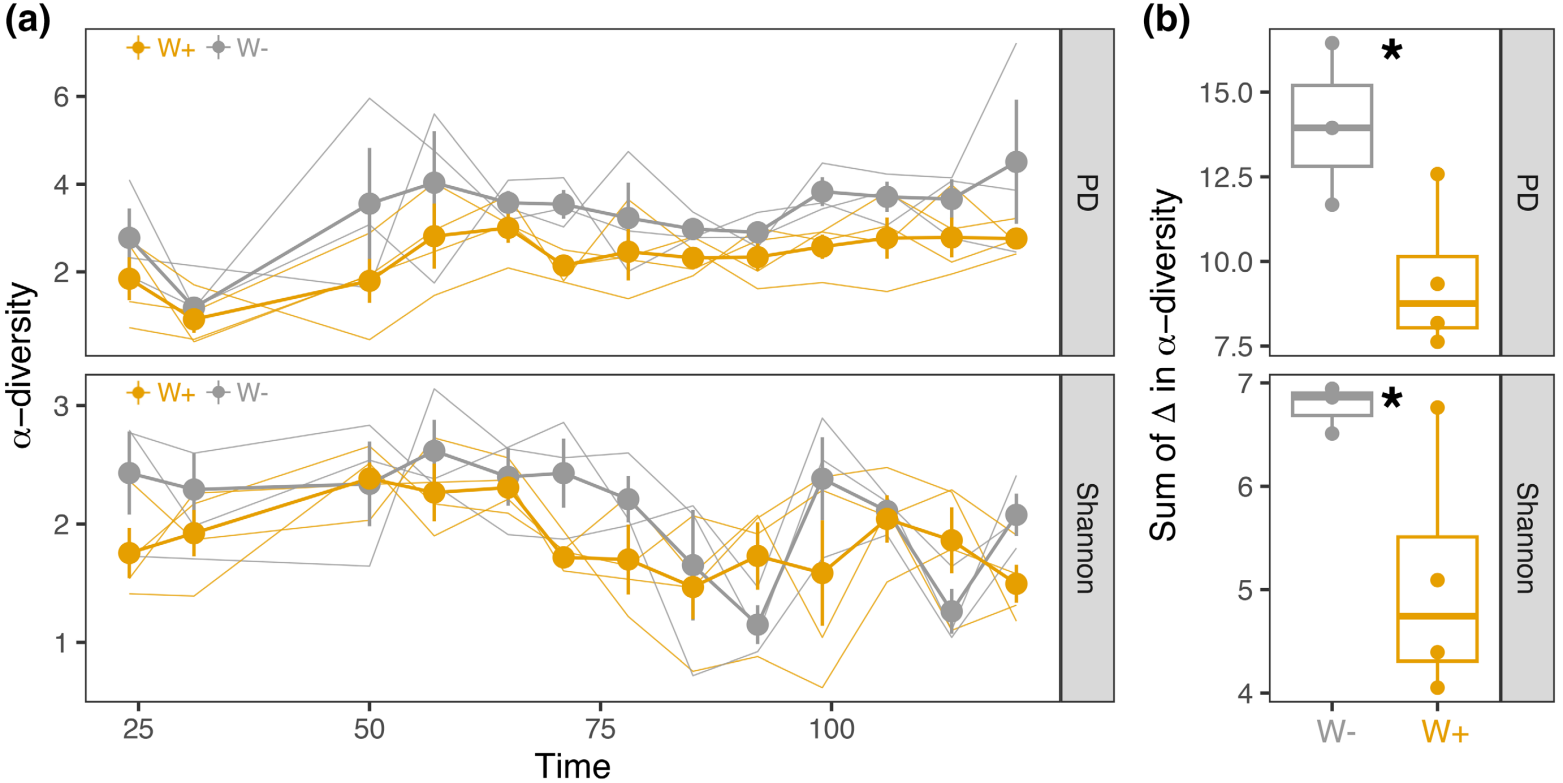
Alpha diversity fluctuates more in *Wolbachia*‐free populations over the season. (a) Alpha diversity measures (PD = Faith's phylogenetic diversity; Shannon diversity) colored by *Wolbachia* status over the growing season. Thicker lines represent the average alpha diversity measure for W+ (orange) and W− (gray) cages. Points represent the mean ± standard error for each timepoint. Thin lines show alpha diversity per cage across the growing season. (b) Summation of the changes in alpha diversity over the season by each cage. Asterisks denote the statistically significant increase in the change of alpha diversity for W− populations.

Indeed, W+ flies exhibited reduced alpha diversity (Figure 3). Shannon diversity fluctuated over the course of the growing season (Figure 3a). First, across all timepoints, Shannon and Faith's phylogenetic diversity (PD) were significantly higher in W− flies (Appendix S1: Figure R1). Furthermore, *Wolbachia* infection significantly impacted both alpha diversity measures over the course of the season (PD: *t* = −3.809, *p* < .001, GAM *R* ^2^ = .457, Appendix S1: Table R1; Shannon: *t* = −2.116, *p* = .037, GAM *R* ^2^ = .218, Appendix S1: Table R2). Over the growing season, W− flies accumulated significantly more changes to both Faith's phylogenetic diversity than W+ flies (Figure 3b, *t* = 2.60, df = 4.24, *p* = .028). Similarly, for Shannon diversity, W− flies accumulated more changes in Shannon diversity (*t* = 2.75, df = 3.28, *p* = .031).

**FIGURE 4:**
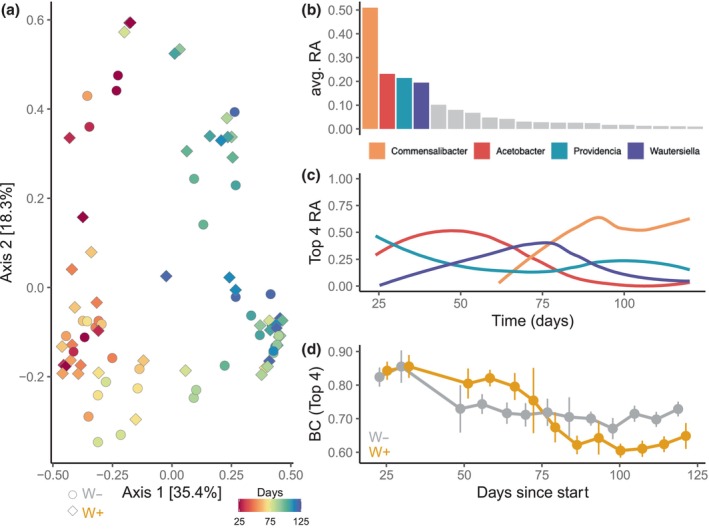
Community composition turnover changes over growing season and interacts with *Wolbachia* status. (a) PCoA plot using Bray–Curtis dissimilarity across all samples and growing season. Color represents time (days since the start), where warm colors represent the summer beginning and cool represent the fall ending. Shape shows *Wolbachia* status. (b) The average relative abundance of different genera across the sampling season. Each bar represents a different genus, but only the top 20 are shown for ease of visualization. *Commensalibacter*, *Acetobacter*, *Providencia*, and *Wautersiella* were most abundant while the other genera (shown in gray) were relatively rare. (c) Temporal dynamics in the four most abundant genera illustrate community turnover. Lines represent the average across all cages for each genus with loess smoothing, and 95% confidence intervals are shaded. (d) Community turnover of the four top genera using Bray–Curtis (BC) dissimilarity for temporal dynamics within cages, by *Wolbachia* status. Points represent mean ± SE. *Wolbachia* interacted significantly with community turnover. W+ populations initially were more dissimilar (i.e., higher BC values), but by the end of the growing season, reduced community turnover and became more similar (i.e., lower BC values).

Differences among flies across the growing season were primarily shaped by growing season and marginally by *Wolbachia* infection status (Figure 4). Principal coordinate analysis using Bray–Curtis dissimilarity (BC) showed that time (i.e., days since the start of experiment) significantly shaped differences between microbiomes (Figure 4a, PERMANOVA: *R* ^2^ = .23, *p* = .001, Appendix S1: Table R3). *Wolbachia* exerted marginal, but significant effects on the microbiome (PERMANOVA: *R* ^2^ = .03, *p* = .001, Appendix S1: Table R3). A similar trend was observed using Unifrac distance, though with *Wolbachia* explaining slightly more variance (Appendix S1: Figure R2, *R* ^2^ for Wolbachia = .07, Appendix S1: Table R4).

To quantify community turnover (i.e., beta diversity), we next assessed the mean BC for each population cage over the growing season. We reasoned that by comparing the change in BC within each cage, we can capture the impact of *Wolbachia* on the dynamics of microbial change within a population (as opposed to across all populations as in Figure 4a). We focused on the top four most abundant genera comprising 83.1% of the microbiome: *Commensalibacter*, *Acetobacter*, *Providencia*, and *Wautersiella*; the other genera were each <10% of the fly microbiome (Figure 4b). As previously described, these four abundant genera showed distinct patterns of change during the growing season (Figure 4c). Notably, *Wolbachia* interacted with seasonality to shape community turnover for the four dominant bacteria (Figure 4d, *Wolbachia* × time interaction: Wald *χ* ^2^ = 12.91, df = 1, *p* = .0003, Appendix S1: Table R5). W+ populations were initially more dissimilar than W−, but became more similar by the end of the season. However, the *Wolbachia* × time interaction was not detected for the complete community (Appendix S1: Figure R3, *Wolbachia* × time interaction: Wald *χ* ^2^ = 1.01, df = 1, *p* = .31, Appendix S1: Table R6). We also extended the analysis for community turnover to the top 10 most abundant bacteria, and we found significant *Wolbachia* × time interactions, but note that the most variance is explained by the top four models (Appendix S1: Figure R4). Taken together, our analysis of community turnover suggests that *Wolbachia* interacts primarily with the dominant taxa within the microbiome and reshapes community turnover.

Through reducing microbiome phylogenetic diversity and turnover (i.e., beta diversity), *Wolbachia* infection likely constrained the ability of the microbiome to respond to the seasonally changing environment. So far, we have considered only microbe–microbe interactions. However, these microbial communities are also changing in the context of the host response to the changing environment.

### 3.3 Interactions between *Wolbachia*, *Commensalibacter*, and fitness‐associated host traits

If interactions between *Wolbachia* and the microbiome influence how the host responds to changing environments, then we would expect to see differences in fitness‐associated phenotypes emerge over the course of the experiment. To test this, we performed periodic phenotyping towards the end of the season for starvation resistance. Starvation resistance reflects the nutritional reserves used in both reproduction and survival in challenging environments. We phenotyped flies at three timepoints and combined with longitudinal microbiome profiling, assessed how interactions between *Wolbachia*, microbiome, and the changing environments shape the response in the fly populations.

**FIGURE 5:**
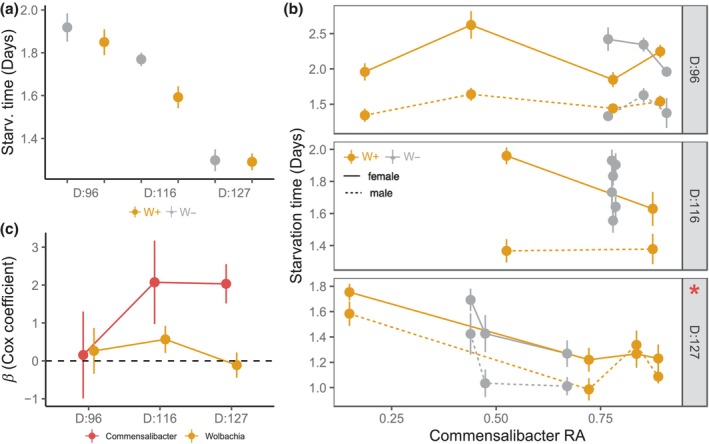
*Commensalibacter* is associated with changes to starvation resistance. (a) Points represent the mean ± SE for time to death (days) at each sampling point. Color represents *Wolbachia* status. Starvation resistance decreased over the growing season. (b) Points represent the mean ± SE time to death for each cage time. Color represents *Wolbachia* status, with solid lines for females and dotted lines for males. For all timepoints, males starved more quickly than females. At Day 96 (*N*: W+ = 82, W− = 77), neither *Wolbachia* nor *Commensalibacter* were associated with changes in starvation time. At Day 116 (*N*: W+ = 59, W− = 92), there was also no statistically significant effect of *Wolbachia* or *Commensalibacter*. At Day 127 (*N*: W+ = 124, W− = 86), the relative abundance of *Commensalibacter* was associated with decreased starvation resistance (red asterisk). (c) Model estimates (*β*) ± SE from Cox hazard models over the three sampling points that summarizes data shown in b. The effects of *Wolbachia* fluctuated, but *Commensalibacter* tended to increase in importance over the growing season.

Starvation resistance varied over the season, with lower starvation resistance at the end of the growing season (Figure 5a). For the microbiome, we focused on the effects of the most frequent bacterium found at the three timepoints and the only bacterium present in all populations, *Commensalibacter*. We note that the relative abundance of *Commensalibacter* was not directly measured from the flies assayed for starvation time (see Section 2), and thus reflects population‐level associations. For the first timepoint (Day 96), only sex affected starvation resistance, where males starved twice as fast as females (Figure 5b, *β*_sex_ = 2.54 ± 0.31 SE, *p* < .0001, Appendix S1: Table R7). Similarly, at the next timepoint (Day 116), only sex affected starvation resistance, with moderate effects of *Commensalibacter* (Figure 5b, *β*_*Commensalibacter*_ = 2.08 ± 1.10 SE, *p* = .06, Appendix S1: Table R8). However, at the final timepoint (Day 127), *Commensalibacter* relative abundance, but not *Wolbachia* status, was significantly associated with variation in starvation resistance (Figure 5b, Appendix S1: Table R9). *Commensalibacter* relative abundance was negatively associated with starvation resistance (*β* = 2.03 ± 0.452 SE, *p* < .0001). Overall, the relative importance of the relationship between *Commensalibacter* and starvation resistance shifted over the growing season (Figure 5c).

**FIGURE 6:**
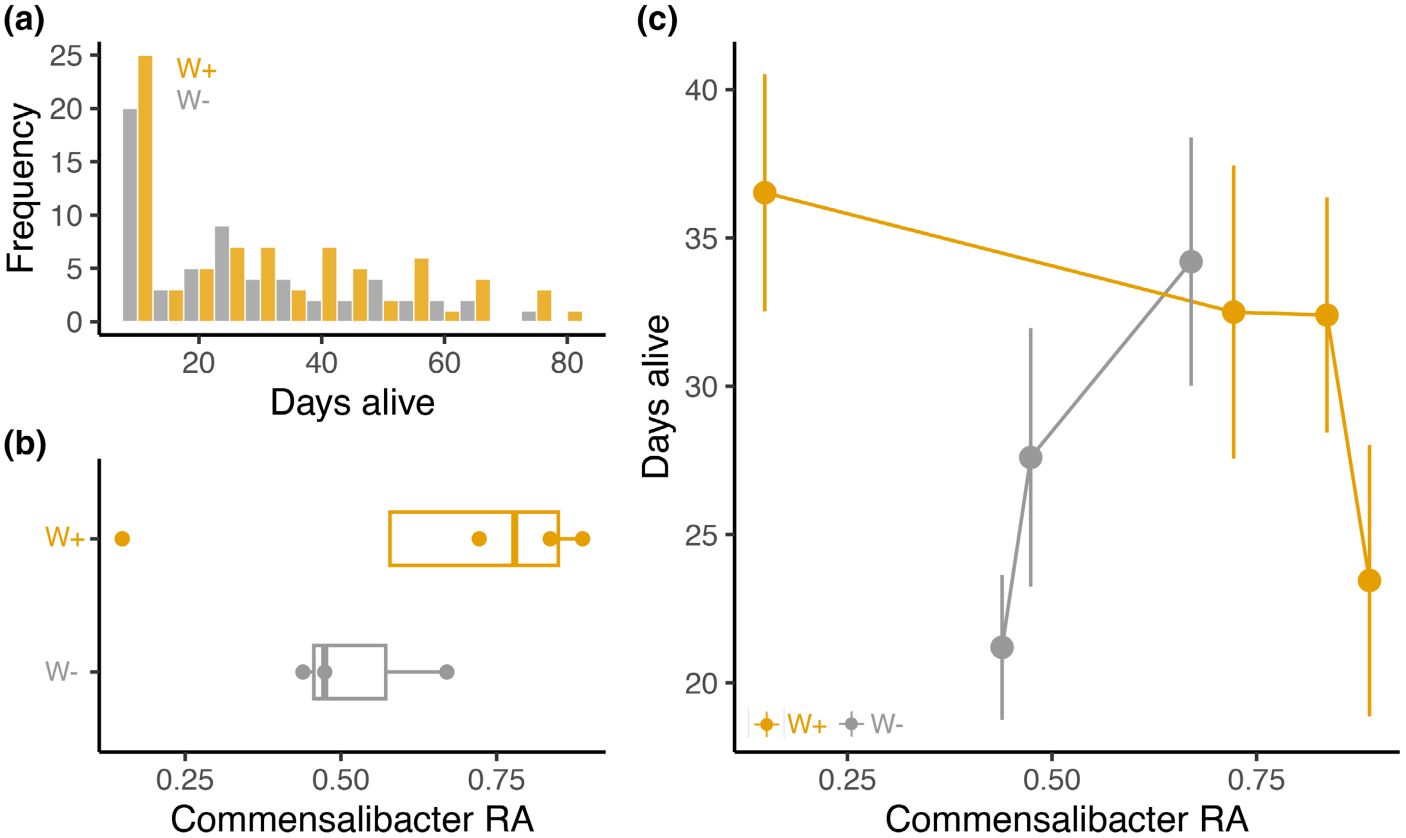
*Wolbachia* and *Commensalibacter* interact to shape lifespan at the end of the growing season. (a) Histogram showing lifespan from the individual females, colored by *Wolbachia* status (*N*: W+ = 79, W− = 60). (b) Boxplots showing relative abundance of *Commensalibacter* from the preceding microbiome sampling point (Day 120). (c) *Commensalibacter* significantly impacted lifespan, but the effect depended on *Wolbachia* status. Points represent mean lifespan ± SE for each cage, colored by *Wolbachia* status.

Finally, at the end of the season (Day 127), we measured lifespan in individual females from each population. While 30% of W+ and 24% of W− females died within the first sampling point (11 days for the first period; after Day 11, checked every 3–4 days until all died), lifespan ranged from 12 to 82 days (Figure 6a). For the microbiome, we examined the relative abundance of the most frequent and only bacterium found across all populations, *Commensalibacter*. *Commensalibacter* did not differ between W+ and W− populations (Figure 6b, Kruskal–Wallis *χ* ^2^ = 1.125, df = 1, *p* = .29). However, significant interactions between *Wolbachia* and *Commensalibacter* were associated with lifespan (Figure 6c, *β* = 3.43 ± 1.52 SE, *p* = .024, Appendix S1: Table R10). For W+ flies, high *Commensalibacter* relative abundance at the point of collection in the field was associated with shorter lifespans, while for W− flies, high *Commensalibacter* was associated with longer lifespans. Together, the interaction between the dominant bacteria in the microbiome, *Commensalibacter*, interacted with *Wolbachia* to shape fitness‐associated traits at the end of the growing season.

## 4 DISCUSSION

Here, we examined how interactions between vertically transmitted and environmentally acquired bacteria shape adaptation to seasonally changing environments in *D. melanogaster*. The analysis of temporal patterns in the fly microbiome (Figure 2) suggests that *Wolbachia* reduced microbial phylogenetic diversity, altered temporal patterns of Shannon diversity, and constrained community turnover (Figures 3 and 4). Furthermore, microbe–microbe interactions between *Wolbachia* and the microbiome were associated with changes in fitness‐associated traits (Figures 5 and 6). The effects of the dominant bacterium, *Commensalibacter*, on starvation resistance varied and depended on *Wolbachia* infection over the growing season, but the interaction differentially shaped lifespan at the end of the season. Next, we discuss how our results provide insights into the influence of complex interactions between *Wolbachia* and the microbiome on host seasonal evolution.

### 4.1 *Wolbachia* constraint on flexibility in the environmentally acquired microbiome

W+ flies had lower phylogenetic diversity and altered temporal patterns of Shannon diversity over the growing season compared to W− flies (Figure 3). Additionally, *Wolbachia* also reduced community turnover by decreasing Bray–Curtis dissimliarity (Figure 4d). The most abundant bacteria responded strongly to *Wolbachia* compared to the total community, suggesting that dominant bacteria potentially regulate or are the most responsive to the interaction between *Wolbachia* and the microbiome. Together, this suggests the presence of *Wolbachia* changes the propensity for microbial change in response to the seasonally changing environment.

The mechanisms underlying *Wolbachia* interactions with other bacteria are poorly understood. *Wolbachia* has been shown to have conflicting effects on Acetobacteraceae, either suppressing (Simhadri et al., 2017) or increasing (Ye et al., 2017) its abundance. The interaction is likely regulated through indirect mechanisms as *Wolbachia* do not infect the lumen cells where most other bacteria reside (Simhadri et al., 2017). *Wolbachia* often interacts with the immune system when protecting against viral pathogens (Chrostek et al., 2013; Teixeira et al., 2008), but has limited effects on protection against bacterial pathogens (Rottschaefer & Lazzaro, 2012; Simhadri et al., 2017; Wong et al., 2011). This suggests that the immune system is not directly involved in regulating the interaction between *Wolbachia* and the environmentally acquired microbiome.

Temperature may however contribute to mediating the temporal dynamics of different bacteria. *Wolbachia*, like other intracellular bacteria, are thermally sensitive, with extreme temperatures exerting negative effects on intracellular bacteria and host fitness (Corbin et al., 2017; Wernegreen, 2012). In *Drosophila*, both high (>28°C) and low (<20°C) temperatures decrease *Wolbachia* abundance and phenotypic effects (e.g., pathogen blocking and reproductive manipulations) in laboratory settings (Chrostek et al., 2021; Hague et al., 2021; Hoffmann et al., 1986; Kriesner & Hoffmann, 2018). Indeed, the proportion of flies infected with *Wolbachia* decreased after the summer but increased back to 100% by the end of the season (Figure 1c). We were unable to measure *Wolbachia* titer in the flies sampled here to understand the impact on fly physiology, but this would be one axis of variation that would be important to incorporate in future studies. Furthermore, because we did not investigate host population genetics, we cannot exclude the possibility that changes in *Wolbachia* infection frequency were due to invasion by W− flies from another cage and/or changing competitive dynamics between W+ or W− flies within a cage due to the impacts of *Wolbachia* on fly physiology. For the environmentally acquired portion of the fly microbiome, however, bacteria are commonly cultured at 30°C (Koyle et al., 2016), with *Providencia* as high as 37°C (Rottschaefer & Lazzaro, 2012), suggesting these bacteria are more tolerant of temperature variation than *Wolbachia*. Reduction in microbiome phylogenetic diversity and altered patterns of temporal change may result from differential growth across bacterial species and the consequences of *Wolbachia* sensitivity to the full range of temperatures experienced over the growing season. Taken together, our results suggest that environmental variation can alter host–microbe interactions through complex responses to abiotic (e.g., temperature) and biotic factors (e.g., microbe–microbe interactions).

While more work is necessary to identify the specific mechanisms underlying *Wolbachia*‐microbiome interactions, our results highlight how *Wolbachia* infection may potentially constrain microbial phylogenetic diversity and alter temporal trends in community turnover. However, assessing potential constraints on microbial diversity requires better understanding of temporal variation in the microbiome. Developing statistical and theoretical approaches to handle time series data in the microbiome is an active area of research (Coenen et al., 2020; Fink et al., 2022; Grieneisen et al., 2023). Nearly all current approaches require filtering the dataset under different criteria and using different community‐level metrics to essentially define the core microbiome. Defining the core microbiome is also nontrivial (Neu et al., 2021; Risely, 2020). Here, we used alpha and beta diversity measures to show that *Wolbachia* infection altered community dynamics during the growing season. For the beta diversity analyses, we detected a significant effect of *Wolbachia* for the most abundant bacteria (Figure 4, Appendix S1: Figure R4). We note that this approach is correlative; we did not assess experimentally through infection or competition experiments whether *Wolbachia* interacts differently with abundant or rare bacteria. Moving forward, linking these effects of changes in microbial diversity to host fitness will be challenging. For example, it is not clear whether the magnitude or pace of change across the growing season mattered more for fly fitness. Future directions should also focus on understanding the interplay between the dynamics of *Wolbachia* across different seasonal pressures and its consequences for variation of infection status within and between fly populations. Connecting these dynamics will be important for understanding the interactions with different environmentally acquired bacteria and their joint role in shaping potential constraints on microbial diversity and effects on hosts.

### 4.2 *Wolbachia* and microbiome interact to shape fitness‐associated traits in hosts

If the microbiome influences host evolution, host phenotypes should change in response to microbial variation (Henry et al., 2021). In *Drosophila*, both *Wolbachia* and the environmentally acquired microbiome often shape variation for a wide range of phenotypes (Douglas, 2018; Fry et al., 2004; Ikeya et al., 2009; McMullen et al., 2020; Starr & Cline, 2002). By examining changes in fitness‐associated traits over the course of the season, we identified shifts in the relative importance of interactions between *Wolbachia* and the microbiome for hosts in seasonally changing environments.

*Commensalibacter*, but not *Wolbachia*, was significantly associated with starvation resistance, and furthermore, the effects depended on the environmental conditions. We only detected a significant effect of *Commensalibacter* relative abundance at the end of the growing season, though there was a trend for the importance of *Commenalibacter* to increase over time (Figure 5). We did not detect any statistically significant effects of *Wolbachia* on starvation resistance. Others also have not found significant effects of *Wolbachia* on starvation resistance in the laboratory populations of *Drosophila* (Ballard et al., 2008; Harcombe & Hoffmann, 2004). For *Commensalibacter*, higher relative abundance was negatively associated with starvation resistance only for the final timepoint. Starvation resistance in flies is predominantly determined by the amount of lipids stored (Hoffmann & Harshman, 1999; Schwasinger‐Schmidt et al., 2012), and while the effects of *Commensalibacter* on lipid stores are unknown, many other bacteria in the Acetobacteraceae family reduce lipid storage (Chaston et al., 2014; Judd et al., 2018). Other bacteria commonly associated with the fly microbiome, like *Lactobacillus*, typically increase lipid storage and are found in fly populations from colder climates (Walters et al., 2020); however, we did not detect *Lactobacillus* in our study. We note that the association we detected between *Commensalibacter* and starvation resistance is correlative. As the starvation assay would impact the microbiome, we used the preceding microbiome data as our covariate in the statistical model. Future work should assess lipid storage and perform manipulative experiments involving *Commensalibacter* and *Wolbachia* infection to better understand how these two microbes influence starvation resistance.

Lifespan was significantly impacted by interactions between *Wolbachia* and the relative abundance of *Commensalibacter* (Figure 6c). Higher abundance of *Commensalibacter* at the point of collection in the field was predictive of longer lifespans for W− flies, but shorter lifespans for W+ flies. While the mechanism underlying this striking result is unknown, these results combined with the starvation results, may suggest one potential mediator – the insulin/insulin‐like growth factor signaling (IIS) pathway. The IIS pathway helps maintain metabolic homeostasis by shaping the balance between carbohydrate availability and lipid storage, and consequently, life‐history tradeoffs in *Drosophila* and many animals (Giannakou & Partridge, 2007). The microbiome also modulates expression of several genes within the IIS pathway, including insulin receptors (Dobson et al., 2017). However, not all bacteria contribute in the same way; different *Acetobacter* species modulate the activity of key components of the IIS pathway in different ways (Shin et al., 2011). *Wolbachia* also increases insulin signaling in *Drosophila* (Ikeya et al., 2009). Furthermore, many genes within the IIS pathway are highly pleiotropic, and polymorphisms in alleles within the IIS pathway also contribute to variation in life history traits associated with adaptation to ecological differentiation along a latitudinal cline in *Drosophila* (Paaby et al., 2014). Interactions between *Wolbachia* and *Commensalibacter* may have shifted the expression of the IIS pathway that led to the different impacts of *Commensalibacter* for the lifespan of W+ and W− flies.

Interaction between microbes and the IIS pathway is just one possible explanation. Bacteria within the fly gut also affect many different aspects of fly physiology, influencing nutrient availability and regulating the expression of different immune pathways (Lesperance & Broderick, 2020; Newell et al., 2014; Tafesh‐Edwards & Eleftherianos, 2023) that together are likely to impact lifespan. More work examining the effects of interactions between *Wolbachia* and different members of the fly microbiome on host gene expression would be necessary to link functional insight with the effects of *Wolbachia*‐*Commensalibacter* interactions on lifespan. We note that *Commensalibacter* abundance was not directly measured in these flies as the measurement of lifespan would impact the microbiome. Rather, here, we used *Commensalibacter* from the preceding timepoint as a predictor of lifespan. Additional experiments that infect W+ and W− flies with *Commensalibacter* would be necessary to casually link *Wolbachia‐*microbiome interactions to lifespan. Overall, our work suggests that complex interactions between *Wolbachia* and the microbiome may shape how hosts allocate nutrition and shift life‐history strategies to buffer environmental change in host populations in seasonally fluctuating environments.

### 4.3 Implications for microbiome interactions in seasonally evolving populations

Here, *Wolbachia* shaped the seasonal changes in the environmentally acquired microbiome, and together, both affected fitness‐associated traits in the flies. These changes overall highlight the potential for variation in microbe–microbe interactions to shape seasonal evolution in hosts – however, the missing link is whether the microbiome changed the host genomic response to selection (Henry et al., 2021). To understand if the microbiome buffered or changed the host evolutionary trajectory, genomic sequencing is necessary. Comprehensive genomic analyses of fly, *Wolbachia*, and the microbiome will provide deep insights into evolutionary processes shaping seasonal evolution.

Previous work in *Drosophila* has shown how other microbiome manipulations shaped seasonal evolution. In a study where flies were inoculated with either *Acetobacter* or *Lactobacillus*, the different bacteria drove genomic divergence between fly populations in only five generations (Rudman et al., 2019). *Acetobacter* enriched fly genomes for alleles associated with southern populations, where *Acetobacter* is also more common (Walters et al., 2020). Similarly, *Lactobacillus* enriched for alleles associated with northern fly populations. The fly populations in these experiments were all infected with *Wolbachia*, but our flies lacked *Lactobacillus*, so applying these findings to our results is only speculative. Nonetheless, taken together, different microbial communities may lead to different evolutionary trajectories. In a sense, the host genome may be tracking the changes in the microbiome. As *Wolbachia* alters the propensity for change in the microbiome for phylogenetic diversity and community turnover, evolution in the host genome also is likely to change, much like adaptive tracking (Bruijning et al., 2020, 2021). More work is necessary to understand the linkages between host and microbiome evolution, but adaptive tracking may depend on host‐microbe interactions (Bruijning et al., 2021). Adaptive tracking in *Drosophila* can occur during seasonal evolution (Rudman et al., 2022), and potentially in the many organisms that live in temporally fluctuating environments – if the microbiome contributes to adaptive tracking remains an open question. Interactions between *Wolbachia* and the microbiome may have shifted the evolutionary trajectories of the W+ and W− populations in different ways that underlie the phenotypic changes to starvation resistance and lifespan observed here. A combination of deep genomic sequencing, reciprocal transplant, and common garden experiments would be necessary to understand the impacts of *Wolbachia*‐microbiome interactions on adaptive tracking and host evolutionary trajectories.

Incorporating microbiome interactions with *Wolbachia* adds additional complexity to an already complex system. However, our results provide insights into how the microbiome may modulate the fitness effects of *Wolbachia* on their host. Mismatches between microbes and the environment may be exacerbated by *Wolbachia*, such as the effects of interaction with *Commensalibacter* on lifespan (Figure 6). Understanding these impacts may also have important implications for efforts to deploy *Wolbachia* to combat mosquito‐borne diseases (Iturbe‐Ormaetxe et al., 2011; Utarini et al., 2021). Interventions to supplement the microbiome with better‐matched microbes may help *Wolbachia*‐infected hosts buffer challenging environments, and many effects of *Wolbachia* observed in *Drosophila* often apply to mosquitoes (Iturbe‐Ormaetxe et al., 2011; Lindsey et al., 2018; Moreira et al., 2009). The physiological consequences of *Wolbachia*‐microbiome interactions may also impact the competitive dynamics between W+ and W− populations. If W+ populations experience a cost of increasing abundance of a particular microbe, as we observed for lifespan, this may make it more difficult for *Wolbachia*‐infected insects to establish as they would be outcompeted by *Wolbachia*‐uninfected insects. As millions of mosquitoes are needed for these *Wolbachia*‐mediated controlled efforts (Crawford et al., 2020; Hoffmann et al., 2011), even moderate improvements to survival by the microbiome may help substantially increase the efficacy of *Wolbachia* introductions in efforts to reduce vector‐borne disease.

In conclusion, when the microbiome changes in seasonally changing environments, *Wolbachia* may modulate effects on fitness‐associated traits in the host. While many questions remain, this study contributes to a growing body of literature utilizing the rewilding of laboratory model systems to uncover how eco‐evolutionary processes in host‐microbe interactions (Hird, 2017; Lin et al., 2020; Rosshart et al., 2017, 2019; Samuel et al., 2016). Future work that links host, microbiome, and their interactions will provide fundamental insights into host‐microbe evolution as well as novel solutions for applied challenges in public health.

## AUTHOR CONTRIBUTIONS

**Lucas P. Henry:** Conceptualization (equal); data curation (lead); formal analysis (lead); funding acquisition (equal); investigation (lead); methodology (lead); supervision (equal); visualization (lead); writing – original draft (lead); writing – review and editing (equal). **Michael Fernandez:** Investigation (supporting); methodology (supporting); writing – review and editing (supporting). **Scott Wolf:** Investigation (supporting); methodology (supporting); writing – review and editing (supporting). **Varada Abhyankar:** Investigation (supporting); methodology (supporting); writing – review and editing (supporting). **Julien F. Ayroles:** Conceptualization (equal); funding acquisition (equal); project administration (lead); supervision (equal); writing – review and editing (supporting).

## FUNDING INFORMATION

LPH was supported by NSF‐GRFP under grant DGE1656466 and National Institutes of Health (NIH) grants GM124881 to JFA.

## CONFLICT OF INTEREST STATEMENT

The authors declare no conflict of interest.

## Supporting information


Appendix S1

## Data Availability

Data are currently available on Dryad DOI: https://doi.org/10.5061/dryad.547d7wmg3. Dryad repository includes fastq files, ASV tables, taxonomy assignment, phenotyping data, and code used to analyze the data.
