# Supplementary material for: Wolbachia impacts microbiome diversity and fitness‐associated traits for Drosophila melanogaster in a seasonally fluctuating environment
Source: Ecol Evol. 2024 Jul 22;14(7):e70004. doi: 10.1002/ece3.70004 (PMC11262851; doi:10.1002/ece3.70004)
Supplement: Supplementary file 1 — Appendix S1 [file ECE3-14-e70004-s001.docx]

Supp. Table M1: Primer sequences used. For the amplicon sequencing primers (16S_27_F and 16S_338_R), the dash separates the Illumina adapters from the 16S rRNA locus.

| **Primer** | **Sequence** |
| --- | --- |
| 16S_27_F | TCGTCGGCAGCGTCAGATGTGTATAAGAGACAG-AGAGTTTGATCMTGGCTCAG |
| 16S_338_R | GTCTCGTGGGCTCGGAGATGTGTATAAGAGACAG-TGCTGCCTCCCGTAGGAGT |
| 16S_wolb_F | TTGTAGCCTGCTATGGTATAACT |
| 16S_wolb_R | GAATAGGTATGATTTTCATGT |
| COI _F | GTAATTGTAACTGCACATGCTT |
| COI_R | ATTCCTAAAGAACCAAAAGTTTC |

Supp. Table R1: Generalized additive model results for Faith’s phylogenetic diversity (PD). Asterisks denote significance.

| **GAMM: log10(PD) ~ Wolbachia + s(Time) + (1\|cage)** | | | | |
| --- | --- | --- | --- | --- |
|  | **Estimate** | **Std. Error** | **t value** | **Pr (> \|t\|)** |
| (Intercept) | 0.4947 | 0.0329 | 15.029 | < 2e-16 *** |
| Wolbachia | -0.167 | 0.0438 | -3.809 | 0.00026 *** |
| **Smoothed terms** | **edf** | **Ref.df** | **F** | **Pr(>F)** |
| s(Time) | 6.196 | 6.196 | 8.315 | 1.25e-06 *** |

Supp. Table R2: Generalized additive model results for Shannon diversity. Asterisks denote significance.

| **GAMM: Shannon ~ Wolbachia + s(Time) + (1\|cage)** | | | | |
| --- | --- | --- | --- | --- |
|  | **Estimate** | **Std. Error** | **t value** | **Pr (> \|t\|)** |
| (Intercept) | 2.0970 | 0.0826 | 25.388 | < 2e-16 *** |
| Wolbachia | -0.2330 | 0.1101 | -2.116 | 0.0374 * |
| **Smoothed terms** | **edf** | **Ref.df** | **F** | **Pr(>F)** |
| s(Time) | 3.815 | 3.815 | 5.54 | 0.00143 ** |

Supp. Table R3: PERMANOVA results for Bray-Curtis dissimilarity between Wolbachia status and time (days since start of experiment). Asterisks denotes significance.

| **PERMANOVA: Bray-Curtis ~ Wolbachia + Time + (strata = cage)** | | | | | |
| --- | --- | --- | --- | --- | --- |
|  | **Df** | **SumsOfSqs** | **F.Model** | **R^2^** | **Pr(>F)** |
| Wolbachia | 1 | 0.6745 | 2.7648 | 0.0245 | 0.001 *** |
| Time | 1 | 6.3992 | 26.1962 | 0.2319 | 0.001 *** |
| Residuals | 84 | 20.5196 |  | 0.7436 |  |
| Total | 86 | 27.5943 |  | 1 |  |

Supp. Table R4: PERMANOVA results for Unifrac distance between Wolbachia status and time (days since start of experiment). Asterisks denote significance.

| **PERMANOVA: Unifrac ~ Wolbachia + Time + (strata = cage)** | | | | | | |
| --- | --- | --- | --- | --- | --- | --- |
|  | **Df** | **SumsOfSqs** | **F.Model** | **R^2^** | **Pr(>F)** |  |
| Wolbachia | 1 | 1.0670 | 7.1892 | 0.0698 | 0.001 *** |  |
| Time | 1 | 1.7449 | 11.7542 | 0.1142 | 0.001 *** |  |
| Residuals | 84 | 12.4675 |  | 0.8160 |  |  |
| Total | 86 | 15.2791 |  | 1 |  |  |

Supp. Table R5: Fixed effects for community turnover (mean Bray-Curtis dissimilarity by each population) over the growing season for the top four abundant bacteria. Significance was evaluated using Type III Wald F tests with Kenward-Rogers degrees of freedom. Asterisks denotes significance.

| **Model: log10(BC.top4) ~ Time * Wolbachia + (1\|cage)** | | | | | | |
| --- | --- | --- | --- | --- | --- | --- |
|  | **Estimate** | **Std. Error** | **df** | **t value** | **Wald X^2^** | **Pr (>X^2^)^b^** |
| (Intercept) | -0.0511 | 0.0135 | 36.2231 | -3.796 | 14.413 | 0.0002 *** |
| Time | -0.0012 | 0.0001 | 74.3160 | -8.650 | 74.827 | < 2.2e-16 *** |
| Wolbachia | -0.0329 | 0.0135 | 36.2231 | -2.439 | 5.950 | 0.0147 * |
| Time x Wolbachia | 0.0005 | 0.0001 | 74.3160 | 3.709 | 13.759 | 0.0002 *** |
|  | | | | | | |

Supp. Table R6: Fixed effects for community turnover (mean Bray-Curtis dissimilarity by each population) over the growing season for the complete microbiome. Significance was evaluated using Type III Wald F tests with Kenward-Rogers degrees of freedom. Asterisks denotes significance.

| **Model: log10(BC.all) ~ Time * Wolbachia + (1\|cage)** | | | | | | |
| --- | --- | --- | --- | --- | --- | --- |
|  | **Estimate** | **Std. Error** | **df** | **t value** | **Wald X^2^** | **Pr (>X^2^)^b^** |
| (Intercept) | -0.0408 | 0.0128 | 39.2678 | -3.188 | 10.1615 | 0.0014 *** |
| Time | -0.0011 | 0.0001 | 78.1758 | -7.738 | 59.883 | 1.01e-14 *** |
| Wolbachia | -0.0121 | 0.0128 | 39.2678 | -0.948 | 0.8994 | 0.3429 |
| Time x Wolbachia | 0.0002 | 0.0001 | 78.1758 | 1.516 | 2.2991 | 0.1295 |

Supp. Table R7: Day 96 starvation resistance. Summary statistics from Cox mixed-effects model fit by maximum likelihood. Asterisks denote significance. Model terms are shown in header.

| **Model: Starvation time ~ Wolbachia * sex + Commensalibacter + (1\|cage) + (1\|imaging plate/arena) + (1\|camera)** | | | | | |
| --- | --- | --- | --- | --- | --- |
|  | **Coefficient** | **Hazard (exp(coef))** | **SE** | **Z** | **p** |
| Wolbachia | 0.2645 | 1.3028 | 0.6030 | 0.44 | 0.66 |
| Sex | 2.5490 | 12.7943 | 0.3150 | 8.09 | 5.6e-16*** |
| Commensalibacter | 0.1549 | 1.1675 | 1.1471 | 0.14 | 0.89 |
| Wolbachia * sex | -0.2198 | 0.8027 | 0.3718 | -0.59 | 0.55 |

Supp. Table R8: Day 116 starvation resistance. Summary statistics from Cox mixed-effects model fit by maximum likelihood. Asterisks denote significance. Model terms are shown in header.

| **Model: Starvation time ~ Wolbachia * sex + Commensalibacter + (1\|cage) + (1\|imaging plate/arena) + (1\|camera)** | | | | | |
| --- | --- | --- | --- | --- | --- |
|  | **Coefficient** | **Hazard (exp(coef))** | **SE** | **Z** | **p** |
| Wolbachia | 0.5666 | 1.7619 | 0.3571 | 1.59 | 0.11 |
| Sex | 1.1146 | 3.0483 | 0.2989 | 3.73 | 0.0002*** |
| Commensalibacter | 2.0741 | 7.9570 | 1.1002 | 1.89 | 0.059 |
| Wolbachia * sex | 0.9118 | 2.4888 | 0.4781 | 1.91 | 0.057 |

Supp. Table R9: Day 127 starvation resistance. Summary statistics from Cox mixed-effects model fit by maximum likelihood. Asterisks denote significance. Model terms are shown in header.

| **Model: Starvation time ~ Wolbachia + sex + Commensalibacter + (1\|cage) + (1\|imaging plate/arena) + (1\|camera)** | | | | | |
| --- | --- | --- | --- | --- | --- |
|  | **Coefficient** | **Hazard (exp(coef))** | **SE** | **Z** | **p** |
| Wolbachia | -0.1126 | 0.8835 | 0.3327 | -0.34 | 0.73 |
| Sex | 1.0212 | 2.7783 | 0.2768 | 3.69 | 0.0002** |
| Commensalibacter | 2.0331 | 7.6374 | 0.5176 | 3.93 | 8.6e-05*** |
| Wolbachia * sex | -0.5957 | 0.5512 | 0.3539 | -1.68 | 0.092 |

Supp. Table R10: Effects of Wolbachia, Commensalibacter, and their interaction (Comm. x Wolb.) on lifespan at the end of the growing season using Cox mixed-effects model fit by maximum likelihood. Asterisks denote significance.

| **Model: Lifespan ~ Wolbachia * Commensalibacter + (1\|cage) + (1\|tube)** | | | | | |
| --- | --- | --- | --- | --- | --- |
|  | **Coefficient** | **Hazard (exp(coef))** | **SE** | **Z** | **p** |
| Wolbachia | -2.1125 | 0.1209 | 0.8464 | -2.50 | 0.013* |
| Commensalibacter | -2.8526 | 0.0577 | 1.4527 | -1.96 | 0.050 |
| Comm. x Wolb.. | 3.4333 | 30.9794 | 1.5220 | 2.26 | 0.024* |

|  |  |  |  |  |  |
| --- | --- | --- | --- | --- | --- |

Supp. Fig. M1: A) Traces from the data loggers in the cages. Two data loggers failed over the course of the experiment and are not shown here. The shaded area represents the range of temperature from each cage, while the line represents the mean temperature.

Supp. Fig. M2: A) Bar plots showing the total *Wolbachia* reads observed in each cage across all sampling points. Bars are colored by *Wolbachia* status, and status is further denoted below each cage label. C7 was removed as it converted (“Conv”) from W- to W+. B) Table showing the total abundance of *Wolbachia* reads detected across all timepoints.

Supp. Fig. M3: PCoA showing difference between samples treated with proteinase K. Each point represents a sample, colored by prot-K treatment. There was no significant difference in beta-diversity (PERMANOVA: Bray-Curtis ~ protK, F_1,212_ = 1.71, p = 0.06).

Supp. Fig. M4: Rarefaction curves for all samples. Each line represents a sample, colored by Wolbachia status. Black line at x = 1000 to show that most communities reached a plateau in ASV richness by a read depth of 1000 reads.

Supp. Fig. M5: Relative abundance of bacteria for each cage at the three phenotyping timepoints. Orange bars on the x-axis show the W+ cages, while grey bars show the W- cages. Colors represent bacteria. Genera with <5% were removed for visualization.

Supp. Fig. M6: Contribution of Commensalibacter relative abundance to beta-diversity for starvation timepoint #1. Each point represents a cage. Points are colored by the Commensalibacter relative abundance. A) Correlation between Commensalibacter and Axis.1 shows that Commensalibacter is strongly correlated with PCoA Axis 1 (estimate = -1.1945, F_1,5_ = 176.8, p = 4.30e-05, adj. R^2^ = 0.967). B) Axis.2 is not correlated with Commensalibacter (estimate = 0.042, F_1,5_ = 0.045, p = 0.8411).

Supp. Fig. M7: Contribution of Commensalibacter relative abundance to beta-diversity for starvation timepoint #2. Each point represents a cage. Points are colored by the Commensalibacter relative abundance. A) Correlation between Commensalibacter and Axis.1 shows that Commensalibacter is strongly correlated with PCoA Axis 1 (estimate = -1.249, F_1,5_ = 73.99, p = 0.0004, adj. R^2^ = 0.924). B) Axis.2 is not correlated with Commensalibacter (estimate = 0.157, F_1,5_ = 0.1215, p = 0.7416).

Supp. Fig. M8: Contribution of Commensalibacter relative abundance to beta-diversity for starvation timepoint #3. Each point represents a cage. Points are colored by the Commensalibacter relative abundance. A) Correlation between Commensalibacter and Axis.1 shows that Commensalibacter is strongly correlated with PCoA Axis 1 (estimate = -0.9931, F_1,5_ = 13.31, p = 0.0148, adj. R^2^ = 0.6724). B) Axis.2 is not correlated with Commensalibacter (estimate = 0.3269, F_1,5_ = 1.309, p = 0.304).

Supp. Fig. R1: Differences in alpha-diversity measures between W+ and W- populations across all timepoints. Faith’s phylogenetic diversity (PD) was significantly different (Kruskall-Wallis X^2^ = 17.01, df = 1, p = 3.71e-05. Shannon diversity was significantly higher in W- than W+ populations (one-sided t test, t = 1.94, df = 72.04, p = 0.03).

Supp. Fig. R2: PCoA showing Unifrac distance. Points are colored by time, with warmer colors at the start of the season and cooler colors at the end of the season. Shapes represent Wolbachia status. Seasonality explained significant variance for Unifrac distance (PERMANOVA, R^2^ = 0.11, p = 0.001). Wolbachia exerted significant, but marginal effects on Unifrac distance between samples (PERMANOVA, R^2^ = 0.07, p = 0.001).

Supp. Fig. R3: Comparisons in community turnover in ASVs between the most abundant 4 bacteria (top 4) and the complete community (all). A) Boxplots showing range in values from all calculations. B) For ease of visualization, only the mean BC dissimilarity is shown over time. Variance in the data can be seen in the A panel.

Supp. Fig. R4: A) Relative abundance of bacteria that comprise the top N taxa. Rare represents the remainder of the community not included in the top N taxa. For example, “top04” represents the top four most abundant bacteria at the genera. We label the bacterial genus that was added in the bar. Overall, the top four make up 83% and top 10 make up 95.7% of the microbiome. Adding other genera marginally increases the proportion of the microbiome included in the community turnover analysis. B) Marginal R^2^ (representing variance explained by the fixed effects) from the models analyzing community turnover. Color denotes if the model was significant for the *Wolbachia* x seasonality interaction. Models are ordered by R^2^. Adding additional taxa from the top 10 most abundant bacteria did not qualitatively change the statistical inference; *Wolbachia* interacts with the dominant taxa within the microbiome and reshapes community turnover.
